# Predicting cardiac and pregnancy outcomes in women with adult congenital heart disease using the Anatomic and Physiological (AP) Classification System: How much does physiology matter?^☆^

**DOI:** 10.1016/j.ijcchd.2023.100486

**Published:** 2023-12-07

**Authors:** Richard Kha, Sarah J. Melov, Thushari I. Alahakoon, Adrienne Kirby, Preeti Choudhary

**Affiliations:** a University of Sydney, Sydney Medical School, Sydney, New South Wales, Australia; b Department of Cardiology, Westmead Hospital, Sydney, New South Wales, Australia; c Westmead Institute for Maternal and Fetal Medicine, Westmead Hospital, Westmead, New South Wales, Australia; d Reproduction and Perinatal Centre, Faculty of Medicine and Health, The University of Sydney, New South Wales, Australia

**Keywords:** Congenital heart disease, Cardiac outcomes, Pregnancy outcomes, High-risk pregnancy, Birth weight, Risk stratification

## Abstract

**Background:**

Pregnancy in women with congenital heart disease (CHD) is associated with an increased risk of adverse maternal and fetal events. Despite the physiological impact of CHD on pregnancy, current risk stratification scores primarily consider anatomical lesions. We assessed the performance of the novel American Heart Association Anatomic and Physiological (AP) classification system in predicting adverse maternal cardiac, obstetric and fetal events, and compared it with established risk models.

**Methods:**

This retrospective cohort study enrolled pregnant women with CHD managed by the Westmead Hospital high-risk pregnancy team. Preconception risk stratification scores (AP classification, mWHO classification, CARPREG II and ZAHARA scores) were retrospectively assigned to each pregnancy by an adult CHD cardiologist and compared with the primary outcome measures, which were maternal cardiac, obstetric and fetal complications.

**Results:**

We analysed 176 pregnancies in 120 women with CHD. Maternal cardiac risk significantly increased between AP class 2 and 3 (p = 0.001). Within class 3, higher physiological status correlated with maternal cardiac events (p < 0.001). Increasing AP severity correlated with lower fetal birthweight percentiles (p = 0.003). The AP classification was similar to mWHO at predicting maternal cardiac outcomes (AUC 0.787 vs 0.777, p < 0.001), but the CARPREG II (AUC 0.852, p < 0.001) and ZAHARA scores (AUC 0.864, p < 0.001) had higher discriminatory ability within our cohort.

**Conclusion:**

The AP classification system shows non-inferior preconception maternal cardiac risk prediction compared to current validated scores. Consideration of physiological status has additive predictive value in the most complex patients (Stage III). Prospective, multicenter studies are required for further validation for preconception risk estimation.

## 1 Introduction

Cardiac disease in pregnancy is the leading cause of maternal deaths in high-income countries [1]. In high-resourced countries, with a lower prevalence of rheumatic heart disease, congenital heart disease (CHD) is the most common cardiac abnormality in pregnancy and accounts for approximately 80 % of the disease burden [2]. Improved survival due to advances in the diagnosis and management of CHD has led to an increase in the number of pregnancies in patients with CHD, even in those with complex conditions [3].

Pregnancy in women with adult CHD is associated with an increased risk of adverse maternal and fetal events [4]. Preconception counselling and multi-disciplinary high-risk teams have shown to improve both maternal and fetal outcomes [4]. Accurate risk estimation using preconception risk scores is crucial in decision-making around commencement and continuation of a pregnancy and for medical optimisation, supervision and intervention [5].

Prediction of peripartum risk in patients with CHD is complex. There is heterogeneity of the baseline cardiac and physical functioning of the patient, geographical variations in delivery of antenatal care, other non-cardiac risk factors and fetal factors including the heightened risk of CHD in the offspring of patients with CHD [4,6,7]. Three major risk scores are widely used today in clinical practice, namely the CARPREG II, ZAHARA and modified World Health Organisation (mWHO) risk scores [2,8,9]. These have been derived from different geographic cohorts and are predominantly based on the anatomical cardiac lesion subtype at birth [10]. The CARPREG II score uses four variables to estimate the risk of cardiac events during pregnancy whereas the ZAHARA uses eight variables. Some variables are common to both the CARPREG II and ZAHARA scores such as prior arrhythmia or heart failure but their relative weighting differs based on the cohort analysed [9,11]. The mWHO classification divides patients into four classes based primarily on anatomical complexity. Most studies support the mWHO classification as having the most superior diagnostic performance [2,3,12,13], although a head-to-head comparison of risk scores in CHD patients is limited because variables used for risk stratification differ across each model and the outcomes are defined differently. Existing risk scores do not systematically assess physiological function for each CHD patient situation. The CARPREG II and ZAHARA scores include some granular physiological endpoints such as NYHA class III or prior arrhythmia, however these endpoints are already well known and do not provide a fine assessment of patient physiology. The mWHO classification is largely anatomical and only includes limited physiological variables such as pulmonary hypertension.

In 2018, the American Heart Association/American Association of Cardiology (AHA/ACC) proposed a new set of CHD classification guidelines called the Anatomy and Physiology (AP) classification system. Unlike current risk scores, the AP classification provides a more systematic and objective analysis of physiological performance to the underlying anatomical lesion [14]. The patient's anatomical lesion is graded into three classes based on complexity, while their physiological status is graded into four stages based on incremental variables including different grades of NYHA function, haemodynamic status and arrhythmia. Both the anatomical lesion and physiological status are combined to give a final AP class. Due to the heterogenous nature of CHD, each person's risk of adverse pregnancy outcome differs based on their initial and residual lesion, operative complications and current functional status [4,6,7]. Patients who have the same anatomical lesion but different physiological status may have varied pregnancy outcomes. Unlike current risk scores, the AP system assesses the patient's physiological function in finer detail to enable better clarification between various physiological states. We hypothesise that systematic evaluation of an individual's anatomy and physiology using the AP classification would assist with preconception risk prediction. To date, the AP classification has not been validated for this purpose.

We aim to evaluate the correlation between the AP score and adverse outcomes including maternal cardiac, obstetric and fetal complications. The secondary aim was to compare the performance of this risk score with the established CARPREG II, ZAHARA and mWHO scores.

## 2 Methods

### 2.1 Study design

We retrospectively reviewed medical records of patients who delivered at Westmead Hospital (WMH) between January 1, 2010 and February 28, 2021. Two obstetric databases were used to collate patients over the study period. Data including patient demographics, CHD diagnosis, medical history and peripartum outcomes were collected. Baseline characteristics were extracted: age, parity, body mass index, gestation at pregnancy booking, ethnicity, initial CHD diagnosis, previous surgery for CHD, residual lesions at time of pregnancy, pre-existing arrhythmia, New York Heart Association (NYHA) class, cardiac medications and echocardiographic findings.

Primary outcome measures included maternal cardiac, maternal obstetric and fetal complications. Maternal cardiac complications included mortality within 12 months of delivery, peripartum hospitalisation (including admission to intensive care unit (ICU)), admission with heart failure, arrhythmia and thromboembolic complications. Obstetric complications included presence of pre-eclampsia/eclampsia, haemorrhage and non-cardiac death. Fetal complications included mortality, preterm delivery, low birthweight, admission to neonatal intensive care unit (NICU) and fetal CHD. Birthweight percentiles were determined using the 2020 Australian birthweight centiles charts [15].

Inclusion criteria were adult pregnant women with CHD who were managed by the high-risk obstetric and adult CHD sub-specialists. Exclusion criteria were: (1) patients with CHD who were not managed by the specialist teams, (2) patients who may have had their care outside the hospital, (3) patients aged less than 18 years, (4) those who had an early miscarriage (i.e., <20 weeks of gestation), and (5) patients with incomplete follow-up data. An adult congenital cardiologist, blinded to the outcomes of pregnancy and patient details, retrospectively assigned risk scores based on the AP classification, mWHO, CARPREG II and ZAHARA score for each patient. We then correlated these with maternal cardiac, obstetric and fetal outcomes.

The study was conducted in compliance with the ethical guidelines of the World Medical Association Declaration of Helsinki. It was approved by the Western Sydney Local Health District (WSLHD) Human Research Ethics Committee (2103-08 QA).

### 2.2 Statistical analysis

The data was analysed using SPSS for Windows (Version 25, Armonk, USA). Normality was assessed using the Shapiro-Wilk test. Normally distributed data were expressed as mean ± standard deviation and medians with ranges were reported for data that were not normally distributed. Correlation between AP classes and outcomes were assessed using Fisher's Chi-square test for categorical variables and Spearman's rank-order correlation test for continuous variables. A p-value <0.05 was used in all statistical tests to reduce random error. ROC (receiver operating characteristic) curves were constructed and analysed for each risk score for adverse maternal cardiac, obstetric and fetal events and the area under the curve (AUC) was calculated. To construct the ROC curves, AP class was firstly converted into a numeric value e.g., 1A was given a score of 1.00, 1B was 1.25, 1C was 1.50, 1D was 1.75, 2A was 2.00 and so on. Three ROC curves were then constructed with adverse maternal cardiac, obstetric and fetal complications as a binary outcome variable using SPSS version 21. The ROC curves were graphed in SPSS by plotting sensitivity against (1-specificity) for each pregnancy risk score. The area under the curve (AUC) and its 95 % confidence interval (95 % CI) was calculated to assess the discriminatory performance of each score. A model with AUC >0.75 is considered to have meaningful discriminatory ability.

## 3 Results

### 3.1 Patient demographics

A total of 120 patients and 176 pregnancies were included in the study. Seventy-six pregnancies (43.2 %) involved nulliparous women whereas 100 pregnancies (56.8 %) involved multiparous women. The four most common CHD lesions were simple shunts (44.9 %), left heart obstructive disease (21.0 %), repaired cyanotic CHD (17.0 %) and right heart obstructive lesions (10.2 %). There were 4 pregnancies with pulmonary hypertension, 2 with Marfan syndrome and 1 with Eisenmenger syndrome in the study cohort. One patient had a mechanical valve and had two pregnancies (see Table 1). Table 2 summarises the adverse outcomes assessed per risk score.

**Table 1:** Baseline characteristics of each pregnancy.

	Number of pregnancies, n (%) N = 176
Maternal age, yrs (mean, SD)	29.18 ± 5.70
Parity	
0	76 (43.2)
1	63 (35.8)
2	26 (14.8)
3	6 (3.4)
4	4 (2.3)
6	1 (0.6)
Body mass index at booking, kg/m^2^ (median, range)	24.7 (16.3–43.1)
Twin pregnancies	2 (1.1)
Late pregnancy assessment (first antenatal visit after 20 weeks gestation)	6 (3.4)
Type of CHD	
Shunts (non-Eisenmenger)	79 (44.9)
Left heart obstructive	37 (21.0)
Right heart obstructive	18 (10.2)
Valvular disease	3 (1.7)
Cyanotic CHD	30 (17.0)
Univentricular physiology	3 (1.7)
Aortopathy	2 (1.1)
Eisenmenger or Pulmonary hypertension	4 (2.3)
AP Class	
1A	72 (41.0)
1B	1 (0.6)
1C	5 (2.8)
1D	3 (1.7)
2A	39 (22.2)
2B	15 (8.5)
2C	12 (6.9)
2D	1 (0.6)
3A	15 (8.6)
3B	6 (3.4)
3C	4 (2.3)
3D	3 (1.7)
mWHO class	
I	87 (49.4)
II	50 (28.4)
III	29 (16.5)
IV	10 (5.7)
CARPREG II	
0-1	122 (69.3)
2	17 (9.7)
3	23 (13.1)
4	9 (5.1)
>4	5 (2.8)
ZAHARA	
0–0.50	94 (53.4)
0.51–1.50	56 (31.8)
1.51–2.50	16 (9.1)
2.51–3.50	4 (2.3)
>3.50	6 (3.4)
Number of patients on anticoagulation	9 (5.1)
Number of patients on cardiac medications	11 (6.3)
NYHA functional class before pregnancy (n = 171)	
I	147 (83.5)
II	24 (13.6)
III	2 (1.1)
IV	0
Type II diabetes mellitus before pregnancy	2 (1.1)
Gestational diabetes mellitus	12 (6.9)
Hypertension	4 (2.3)
Smoking	10 (5.7)
Substance abuse during pregnancy	1 (0.6)
History of pre-eclampsia	7 (4.0)

**Table 2:** Adverse outcomes as per assessed risk scores.

Risk stratification system	Number of deliveries, n	Cardiac events, n (%)	Obstetric events, n (%)	Fetal/neonatal events, n (%)
AP class				
1A	70	2 (2.9)	22 (31.4)	19 (27.1)
1B	1	1 (100)	1 (100)	1 (100)
1C	5	0	2 (40)	2 (40)
1D	3	1 (33.3)	1 (33.3)	2 (66.7)
2A	39	2 (5.1)	7 (17.9)	11 (28.2)
2B	15	2 (13.3)	6 (40)	5 (33.3)
2C	12	1 (8.3)	6 (50)	6 (50)
2D	1	0	0	1 (100)
3A	15	1 (6.7)	7 (46.7)	9 (60)
3B	6	2 (33.3)	1 (16.7)	5 (83.3)
3C	4	3 (75)	1 (25)	1 (25)
3D	3	3 (100)	2 (66.7)	3 (100)
mWHO class				
I	86	3 (3.5)	25 (29.1)	21 (24.4)
II	50	4 (8.0)	18 (36)	18 (35)
III	28	7 (25)	10 (35.7)	16 (57.1)
IV	10	4 (40)	3 (30)	10 (100)
CARPREG II				
0-1	121	3 (2.5)	35 (28.7)	38 (31.4)
2	16	1 (6.3)	3 (17.6)	8 (47.1)
3	23	5 (21.7)	9 (41)	13 (56.5)
4	9	5 (55.6)	7 (77.8)	4 (44.4)
>4	5	4 (80)	2 (50)	2 (50)
ZAHARA				
0–0.50	93	1 (1.1)	24 (25.8)	28 (30.1)
0.51–1.50	55	7 (12.7)	20 (36.3)	23 (41.8)
1.51–2.50	16	5 (31.3)	6 (37.5)	11 (68.8)
2.51–3.50	4	2 (50)	4 (100)	1 (25)
>3.50	6	3 (50)	2 (33.3)	2 (33.3)

### 3.2 Correlation between AP classification and pregnancy complications

**Fig. 1:**
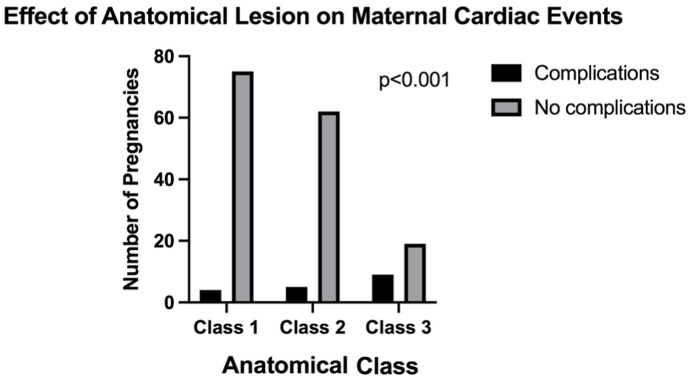
Effect of anatomical class on maternal cardiac outcomes.

The prevalence of maternal cardiac complications increased significantly with increasing anatomical severity of the AP classification system (p < 0.001) (Fig. 1). The greatest increase in cardiac risk was observed between Class 2 and Class 3 (24.6 %; p = 0.001).

**Fig. 2:**
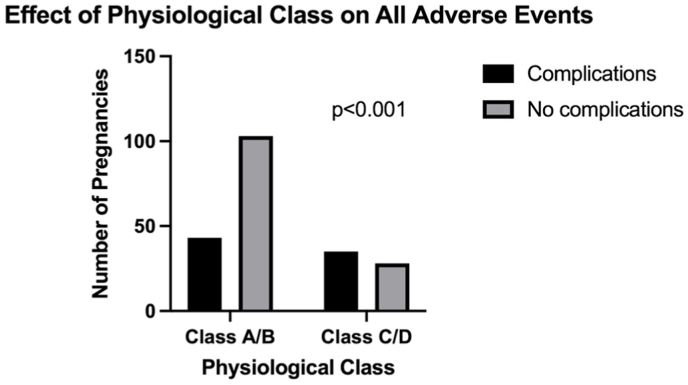
Effect of physiological class on maternal cardiac outcomes.

The effect of physiological complications was assessed by comparing the milder spectrum (A/B class) to the more severe spectrum (C/D class) within each anatomical class (Fig. 2). Overall, patients with worse physiological class had a significantly higher prevalence of both maternal cardiac complications (6.8 % vs. 28.6 %; p = 0.002) and all composite complications (29 % vs. 56 %, p < 0.001).

Patients in Class 3 demonstrated a marked difference in complication rates between low to high physiological severity (14.3 % vs. 85.7 %; p < 0.001). There was no significant effect of physiology on complications within either Class 1 (4.2 % vs. 12.5 %; p = 0.31) or Class 2 (7.4 % vs. 7.7 %; p = 0.97).

The prevalence of obstetric complications was 32.9 % in AP Class 1, 28.4 % in Class 2 and 39.3 % in Class 3. There was no significant correlation between AP class and the occurrence of obstetric complications (p = 0.54). The prevalence of fetal complications was 30.4 % in AP Class 1, 34.3 % in Class 2 and 64.3 % in Class 3. The median birthweight percentile was 53.5 (2.0–99.0) in Class 1, 45.0 in Class 2 (1.0–99.0) and 23.0 in Class 3 (1.0–80.0). There was a weak but statistically significant relationship between the median birthweight percentile and AP class (ρ = −0.239, p = 0.003).

### 3.3 Comparison of AP class with mWHO, CARPREG II and ZAHARA

**Fig. 3:**
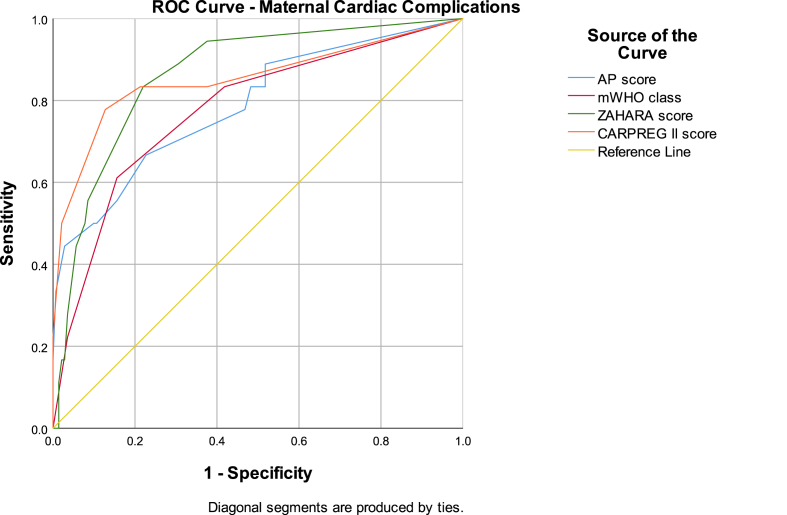
Receiver operating characteristic (ROC) curve for maternal cardiac complications.

**Fig. 4:**
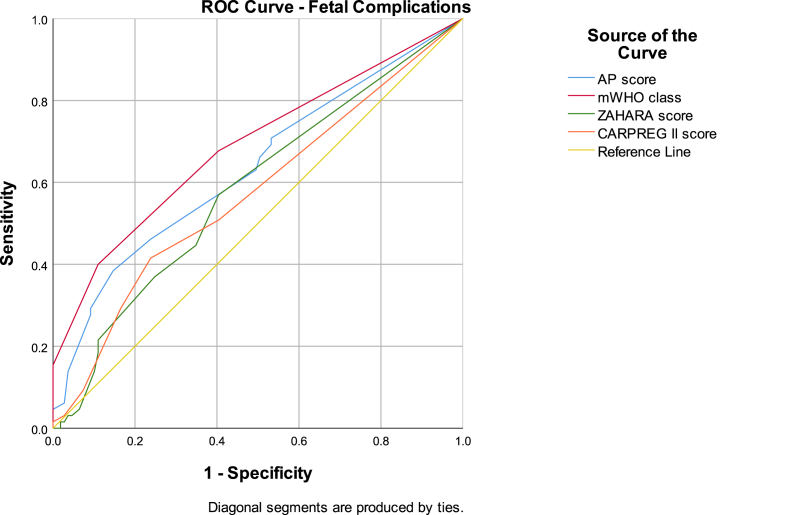
Receiver operating characteristic (ROC) curve for fetal/neonatal complications.

The ZAHARA (AUC 0.864 (95 % CI: 0.779–0.949; p < 0.001) and CARPREG II (0.852 (95 % CI: 0.732–0.973; p < 0.001) scores had the best diagnostic performance for predicting adverse maternal cardiac events. The AP and mWHO classifications were comparable with AUC 0.787 (95 % CI: 0.663–0.911; p < 0.003) and 0.777 (95 % CI: 0.655–0.898; p < 0.001) respectively (see Table 3 and Fig. 3). The risk scores performed poorly for adverse obstetric events with AUC <0.590. Both the AP and mWHO scores demonstrated modest but statistically significant discrimination for fetal adverse events (AUC 0.636 (95 % CI: 0.548–0.723; p = 0.003) and 0.689 (95 % CI: 0.604–0.773; p = 0.001)) respectively (see Table 4 and Fig. 4).

**Table 3:** Receiver operating characteristic (ROC) curve for maternal cardiac complications.

Risk estimation method	AUC	95 % CI	p-value
ACHD-AP classification	0.787	(0.663, 0.911)	<0.001
mWHO classification	0.777	(0.655, 0.898)	<0.001
CARPREG II score	0.852	(0.732, 0.973)	<0.001
ZAHARA score	0.864	(0.779, 0.949)	<0.001

**Table 4:** Receiver operating characteristic (ROC) curve for fetal/neonatal complications.

Risk estimation method	AUC	95 % CI	p-value
ACHD-AP classification	0.636	(0.548, 0.723)	0.003
mWHO classification	0.689	(0.604, 0.773)	<0.001
CARPREG II score	0.573	(0.483, 0.662)	0.108
ZAHARA score	0.583	(0.495, 0.671)	0.066

**Fig. 5:**
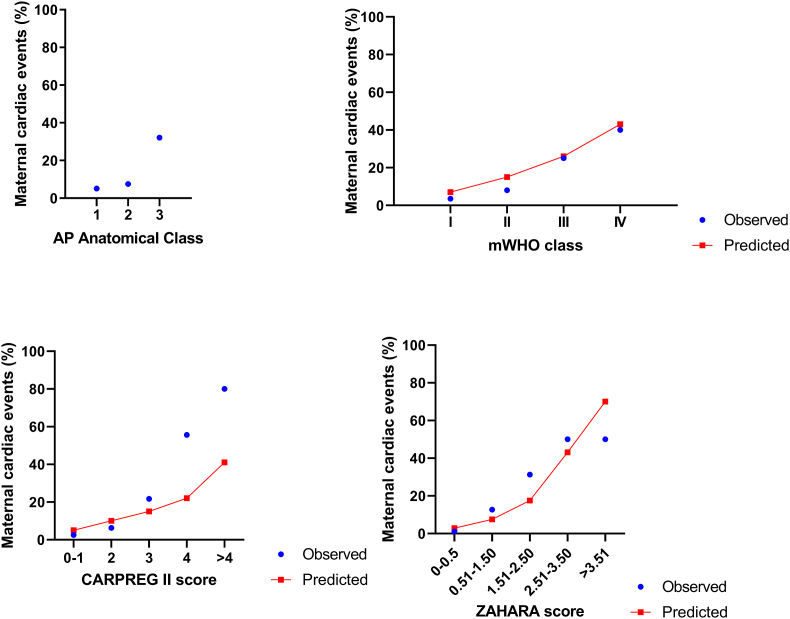
Number of patients, observed and predicted risk for adverse maternal cardiac events for different risk prediction models.

Fig. 5 details the distribution of actual vs. expected maternal cardiac events in our cohort for each risk score. In our cohort, the ZAHARA or CARPREG II risk scores underestimated actual cardiac risk. The modified WHO was more closely matched with adverse outcomes. The AP score correlated well with modified WHO scores.

## 4 Discussion

Our results demonstrate that the AP classification system correlates well with maternal cardiac risk and fetal birthweight. The ZAHARA and CARPREG II scores underestimated risk in the highest AP class. Its performance was comparable to the mWHO scoring system.

Our results are consistent with published data that demonstrates that greater anatomical lesion complexity is associated with adverse maternal cardiac outcomes. Most importantly, our results demonstrated that across all anatomical lesions, impaired baseline physiological function independently correlated with adverse cardiac and total adverse events. The impact of physiology on adverse cardiac outcomes was much greater for pregnant women with complex congenital lesions compared to those with mild lesions. In the most severe anatomical class, physiological function is more clinically relevant than milder anatomical classes as worse physiological function (class C/D) is significantly correlated with adverse maternal cardiac and all adverse outcomes. This also suggests that anatomical lesion is the driver of maternal cardiac outcomes in patients with less severe disease. These findings were not captured by the results from studies evaluating the other risk scores. While other scoring systems demonstrate a unidirectional relationship of the respective scores and risk of adverse outcomes, the AP classification shows that risk estimation is not necessarily unidirectional. For example, class 2C could be worse than class 3B due to the lower physiological and haemodynamic function. One potential application of the AP classification system would be as a two-tier estimation process. The first step would be to determine the anatomical class to first establish the baseline risk, followed by determining the physiological class to further refine the risk before and/or during the course of pregnancy. Hence, we demonstrate the incremental value of using the AP classification system to assess physiological performance for overall risk prediction in CHD patients.

Risk scoring markers are complex and the variable performance of three established risk scores within a real-world cohort of patients at a tertiary centre in a high-resourced country underscores this complexity. The ZAHARA and CARPREG II scores had a greater AUC on ROC curves which may be explained by having a higher proportion of simple CHD i.e. a greater proportion of shunts within our cohort. Clinical risk prediction is more conservative than statistical risk prediction given the consequences of underestimating risk which are far greater than when risk is overestimated. Clinically this would translate to increased surveillance, admission to hospital at a lower threshold and closer monitoring of the patient.

Discussion of pregnancy risk starts to occur in adolescence when clinicians discuss contraception with a patient with CHD. Early understanding of how physiology would impact long-term outcomes are important for long-term decision-making. The ability to use the AP classification through the years would further refine this risk prediction. It is unknown whether changing the physiological state i.e. treating arrhythmia with ablation would affect long-term pregnancy outcomes. Although our data suggests this, future long-term studies involving large cohorts are required to further assess this. Furthermore, unlike anatomical status, an individual's physiological status can alter throughout the course of their pregnancy. With further studies involving larger cohorts of patients, the AP classification can potentially enable clinicians to reclassify patients by assessing their physiological status to detect whether escalation of care is required. Steiner et al. reported a similar finding in their cohort, however physiological status was more strongly predictive of outcomes [16]. Oumbret et al. reported a correlation between AP class and mortality [17], underscoring the importance of assessing both facets in these patients and highlighting the emerging clinical utility of this classification system.

Our findings demonstrated comparable performance between the AP classification system and mWHO classification system in predicting both maternal cardiac risk and fetal adverse outcomes. This is promising because the mWHO score has been shown to have the highest discriminatory power in multiple studies [3,[18], [19], [20]]. However, we did note a lower observed complication rates for each mWHO class compared to the expected complication rates. Our cohort also had a relatively low maternal cardiac complication rate of 10.9 %, compared to published cohorts with a similar distribution of patient complexity [3,9,11,[21], [22], [23], [24], [25]]. This may reflect the lower complication rates among our patients who were managed at targeted high-risk pregnancy centres and received high levels of specialised care.

Furthermore, the ZAHARA and CARPREG II scores displayed better discriminatory ability for maternal cardiac outcomes within our cohort. This can be explained by the fact that commonly used risk scores are derived from different geographic cohorts with varying complexity of CHD and hence may produce varying results when applied to different CHD cohorts [5]. The ZAHARA score is derived from a CHD cohort and is weighted (e.g., mechanical valve automatically scores 4 points) which may explain its better performance in a complex group of patients. ZAHARA score closely approximates maternal cardiac event risk in the majority of our patients, however there was an outlier in the very high risk group of patients (i.e. ZAHARA >3.51). This could be explained by the low prevalence of very high risk patients within our cohort. The CARPREG II score was derived from patients with both congenital and acquired heart disease with ∼25 % of the cohort having no CHD [11]. It is therefore plausible that the higher prevalence of observed complications using the CARPREG II score in our cohort reflects the higher complexity of CHD patients. The variation in performance of multiple scores highlights the complexity of care and that risk scores are a guideline, therefore clinical judgement and frequent patient evaluations remain crucial.

The results in our study were similar to the study by Kim et al. comparing the ZAHARA and mWHO risk prediction scores [21]. The AUC for the ROC curves were similar to our study for the ZAHARA (AUC = 0.80) and mWHO (AUC = 0.77) scores, with the ZAHARA score showing the best discriminatory performance [21]. Another smaller, recent study by Bredy et al. had similar AUC for mWHO (AUC = 0.75) but lower performance for CARPREG II (AUC = 0.65) and ZAHARA (AUC = 0.59) compared to our study [26]. The study also suggested the potential importance of haemodynamic and physiological function in minimising adverse outcomes, which has been explored in our study using the AP classification method. Both studies found that the predicted risk of adverse outcomes was lower than the observed rate, which is consistent with the findings in our study [21,26]. These findings further highlight the challenges of predicting risk due to the heterogeneity of CHD.

Consistent with other studies [5,22], there was no significant correlation between AP class and obstetric outcomes. AP classification was associated with higher risks of fetal events and low fetal birthweight percentiles. This finding is consistent with large scale studies [4,27,28], supporting the hypothesis that abnormal uteroplacental vessel formation and blood flow [29] in CHD patients may lead to adverse fetal outcomes.

### 4.1 Strengths and limitations

This is one of the first studies to independently assess the utility of the AP Classification developed for CHD patients in predicting pregnancy-related outcomes. We included a large number of complex CHD patients with peripartum management via a single high-risk team which ensures relatively consistent obstetric management across the heterogenous cohort.

Selection bias included management at a single tertiary/quaternary level cardiac centre and selection of patients in the public health sector. The patients who were managed in the private health system were not included and may have had different outcomes. The effects of these biases were minimised by including patients from private cardiologists who were managed at the hospital. A higher sample size would have increased numbers within each of the 12 AP classification categories and this is certainly an important future direction of this group. The number of patients in the higher anatomical and physiological severity classes in our study was low. This is primarily due to the fact that women with severe anatomical lesions or physiological dysfunction are not recommended to pursue pregnancy, thus making it difficult to recruit patients in these classes. This is also an issue in other studies comparing various risk prediction scores for adverse outcomes in adult CHD pregnancies. The low patient numbers in higher AP classes may introduce precision error in the outcome measures among each AP class and a larger collaborative study involving multiple centres would be preferred for future validation studies.

## 5 Conclusion

This study showed that the AP classification system has comparable outcomes as the anatomically based mWHO classification system for preconceptual risk assessment. We demonstrate an incremental value of assessing physiological status, especially in the highest risk groups. This may translate to clinical management to improving preconceptual physiological status in patients with severe CHD. Risk assessment in CHD patients is complex and sophisticated requiring integration of both anatomical and physiological factors. Prospective, multi-centre studies are required to validate the use of this classification system in pregnancy.

## Funding

The research did not receive any specific grant from funding agencies in the public, commercial, or not-for-profit sectors.

## CRediT authorship contribution statement

**Richard Kha:** Conceptualization, Investigation, Methodology, Resources, Software, Writing – original draft, Writing – review & editing, Data curation, Formal analysis. **Sarah J. Melov:** Investigation, Methodology, Supervision, Validation, Writing – review & editing. **Thushari I. Alahakoon:** Investigation, Methodology, Supervision, Writing – review & editing. **Adrienne Kirby:** Investigation, Methodology, Software. **Preeti Choudhary:** Conceptualization, Formal analysis, Investigation, Methodology, Supervision, Validation, Writing – original draft, Writing – review & editing.

## Declaration of competing interest

The authors declare that they have no known competing financial interests or personal relationships that could have appeared to influence the work reported in this paper.
